# A robust statistics driven volume-scalable active contour for segmenting anatomical structures in volumetric medical images with complex conditions

**DOI:** 10.1186/s12938-016-0153-6

**Published:** 2016-04-14

**Authors:** Kuanquan Wang, Chao Ma

**Affiliations:** School of Computer Science and Technology, Biocomputing Research Center, Harbin Institute of Technology, Harbin, China

**Keywords:** Volumetric medical image segmentation, Active contour, Robust statistics, Volume scalable, Complex conditions

## Abstract

**Background:**

Accurate segmentation of anatomical structures in medical images is a critical step in the development of computer assisted intervention systems. However, complex image conditions, such as intensity inhomogeneity, noise and weak object boundary, often cause considerable difficulties in medical image segmentation. To cope with these difficulties, we propose a novel robust statistics driven volume-scalable active contour framework, to extract desired object boundary from magnetic resonance (MR) and computed tomography (CT) imagery in 3D.

**Methods:**

We define an energy functional in terms of the initial seeded labels and two fitting functions that are derived from object local robust statistics features. This energy is then incorporated into a level set scheme which drives the active contour evolving and converging at the desired position of the object boundary. Due to the local robust statistics and the volume scaling function in the energy fitting term, the object features in local volumes are learned adaptively to guide the motion of the contours, which thereby guarantees the capability of our method to cope with intensity inhomogeneity, noise and weak boundary. In addition, the initialization of active contour is simplified by select several seeds in the object and/or background to eliminate the sensitivity to initialization.

**Results:**

The proposed method was applied to extensive public available volumetric medical images with challenging image conditions. The segmentation results of various anatomical structures, such as white matter (WM), atrium, caudate nucleus and brain tumor, were evaluated quantitatively by comparing with the corresponding ground truths. It was found that the proposed method achieves consistent and coherent segmentation accuracy of 0.9246 ± 0.0068 for WM, 0.9043 ± 0.0131 for liver tumors, 0.8725 ± 0.0374 for caudate nucleus, 0.8802 ± 0.0595 for brain tumors, etc., measured by Dice similarity coefficients value for the overlap between the algorithm one and the ground truth. Further comparative experimental results showed desirable performances of the proposed method over several well-known segmentation methods in terms of accuracy and robustness.

**Conclusion:**

We proposed an approach to accurate segment volumetric medical images with complex conditions. The accuracy of segmentation, robustness to noise and contour initialization were validated on the basis of extensive MR and CT volumes.

## Background

Active contour models (ACMs) have been studied extensively in recent decades and widely used in image segmentation with promising results [1, 2]. Compared with the classical image segmentation methods, such as region-growing, edge detection and artificial neural network (ANN), ACMs have several desirable advantages. For example, the models can provide smooth and closed contours as segmentation results with sub-pixel accuracy [3], and meanwhile they can easily integrate with various prior knowledge [4]. A comparative study of major ACMs can be found in [5]. Generally, there are two major classes of ACMs: edge-based models [6, 7] and region-based models [8, 9].

Typical edge-based models drive active contour toward the object boundary using image gradient information. These models are very sensitive to noise and weak object boundary. These drawbacks limit their applications for medical images, which typically contain noise induced by the image acquisition process and fuzzy boundary caused by low contrast or partial volume effect [10]. In contrast to edge-based schemes, region-based models have better performances in the presence of noise and weak boundary due to the utilization of certain region descriptors. However, most of the region-based models [11, 12] rely on the assumption of intensity homogeneity in each of the regions that compose the image domain, and therefore they usually fail to segment medical images with non uniform intensity.

In fact, real-world images are often distorted by intensity inhomogeneity and/or noisy weak object boundary. For medical images, such as MR and CT images, imperfect image conditions are usually caused by imperfections of imaging devices or imaging artifacts introduced by the movement of the object being imaged. In particular, due to the effects of non uniform magnetic fields and partial voluming, the intensity inhomogeneity and weak boundary often appear in MR images. Moreover, in order to fully utilize the information given by the volumetric medical images, it needs to segment the volumetric image data directly in three dimension in some circumstance. The acquisition sequences which compose the volumetric image can also introduce intensity inhomogeneity that appears as an intensity variation across the image slices. Accuracy in volumetric medical image segmentation is therefore hard to achieve.

In the past several years, many efforts were put into complex medical image segmentation [13–15], in meeting the variety of needs of clinical diagnosing and therapy, including local region-based, graph-based, atlas-based, etc. Among these methods, local region-based method is a widely used technique for segmentation of complex medical images. Local information can be extracted from local regions of inhomogenous images and be incorporated into the energy functional [16–18]. For example, Li et al. [19] proposed a data fitting energy by approximating image intensities in local regions at a controllable scale and then integrated it into a variational level set formulation for image segmentation. Lankton et al. [20] developed a natural framework that allows any region-based segmentation energy to be re-formulated in a local way and evolve a contour based on local information. This method was later improved by Mille J. [21]. However, common limitations of all these local region-based methods are that they generally are sensitive to initial contour and high levels of noise. Some hybrid methods are also proposed that integrate local region-based level set methods with a good deal of image processing techniques, such as clustering [22], global intensity fitting [23, 24], local statistical function [25], hierarchical voxel analysis in multi-resolution [26], patch-based sparse representation techniques [27] and signed pressure force (SPF) function [28], improving the segmentation performance of local region-based methods. The coefficients of these methods are, however, sometimes difficult to adjust, which limits their practical applications.

As proposed in [29], the bias field accounts for the intensity inhomogeneity, which can be corrected along with tissue segmentation based on an expectation-maximization (EM) algorithm. Therefore this method can deal with intensity inhomogeneity. Some related methods were later proposed in [30, 31], which have certain capability of handling intensity inhomogeneity. However, due to the complicated and non-linear intensity inhomogeneity, these methods may fail to get accurate segmentation results for the images in which the bias fields are hard to estimate.

Graph-based techniques have achieved good performances for natural image segmentation, such as graph cuts [32–34], random walker [35], isoperimetric graph partitioning [36] and normalized Cuts [37], which map the image elements onto a mathematically sound graph, and then segmentation proceeds by the flexible and efficient tools from graph theories [38]. As for the complex medical image segmentation in the presence of imperfect image conditions, Liu et al. [39] and Petersen et al. [40] make use of novel graph-based segmentation methods to extract non-intersecting columns that are applicable for surfaces with high curvature or complex shapes, such as human airway walls. Huang et al. [41, 42] provided a robust graph-based segmentation algorithm to extract breast tumors in ultrasound images with speckles and low contrast. Li et al. [43] developed a graph-theoretic approach to efficient segment object boundaries in volumetric data sets. Song et al. [44] presented an inhomogeneity correction method by adaptively adjusting the edge weights in graph cuts for brain MRI segmentation. However, many popular graph-based segmentation methods are restricted by image size in practice due to the increasingly computational and memory burdens as more nodes and edges are added to the graph. Especially for volumetric medical images, which can contain billions of voxels, segmentation on volumetric graph defined over such large volumes of data would be intractable [45].

As an extension of graph-based methods, atlas-based methods make use of spatial prior information, which can be generated from manual or automated segmentation of training images, to guide the segmentation of the target images [46]. In order to compensate for the potential biases and errors, individual atlases can be further fused as multi-atlas [47–49]. Atlas-based methods often exhibit good performances for many physiological structure segmentation tasks even in the presence of reduced tissue contrast and increased noise [50, 51]. However, they are challenged by the problem of pathology segmentation due to the considerable variation of these pathologies across patients in terms of shape, size, and localization.

Machine learning methods, in particular, random forest (RF) methods have recently enjoyed the increased attentions in the complex medical image segmentation [52, 53]. They are inherently suited for handling a large number of multi-class image data with high image feature dimension, and achieve promising results for some tissue segmentation tasks [54]. For example, Li Wang et al. [55] proposed a RF-based multi-source integration framework for segmentation of infant brain images by fully capturing both local and contextual image information. Tustison et al. [56] incorporated the optimal symmetric multimodal templates into the concatenated random forests for supervised brain tumor segmentation. One of the few drawbacks of the RF-based methods is that an unsophisticated depth of the decision tree will likely lead to under-fitting or over-fitting.

Besides, most of the above-mentioned methods perform segmentation in 2D whose scalability to 3D are not tested. Segmentation algorithms that focus on volumetric data are potentially more efficient and perform better in complex tissue areas [57, 58]. For instance, Gu et al. [59] initialized a 3D active surface model inside the airway regions and thereafter allowed this model to evolve under predefined external and internal forces automatically to reach the airway wall. Ukwatta et al. [60] developed a new coupled min-cut/max-flow formulation for 3D segmentation of the femoral artery lumen and outer wall from black-blood MR images. Yaqub et al. [61] investigated the role of feature selection and weighted voting within the random forest classification framework for 3D volumetric segmentation. Chandra et al. [62] integrated the weighted shape priors into the deformable models for hip joint segmentation in 3D MR images. Jiang et al. [63] proposed a 3D brain tumor segmentation method by learning the population- and patient-specific feature sets of multimodal MR images. Compared with 2D segmentation techniques, more image information can be fed into the 3D segmentation algorithms, and more complex image conditions are imposed at the same time, which makes the 3D segmentation a challenge problem. Furthermore, most of the 3D segmentation methods are designed for segmenting specific anatomical structures and lack the ability of segmenting multi-class structures.

Recently, the probability distribution function (PDF) based description has attracted rapidly growing interest [64, 65]. This approach introduces alternative similarity measurements into the level set framework base on PDFs extracted from the regions on the two sides of the evolving contour [66, 67]. This strategy has been proven to be efficient for describing the images with complex local information [68].

### Related works

#### The region-scalable fitting (RSF) model

In order to cope with intensity inhomogeneity, Li et al. [19, 69]. proposed the RSF model by utilizing the image intensity information in local regions. By introducing a kernel function, they defined the following energy functional:

1 $$\begin{aligned} \varepsilon^{Fit} (C,f_{1} ,f_{2} ) &= \lambda_{1} \int {\left[ {\int_{outside(C)} {K_{\sigma } (x - y)\left| {I(y) - f_{1} (x)} \right|^{2} dy} } \right]} dx \\ & \quad + \lambda_{2} \int {\left[ {\int_{inside(C)} {K_{\sigma } (x - y)\left| {I(y) - f_{2} (x)} \right|^{2} dy} } \right]dx} \\& \quad + \nu |C| \end{aligned}$$

where *K*_*σ*_ is a Gaussian kernel with standard deviation *σ*, *f*_1_ and *f*_2_ are two spatially varying fitting functions that locally approximate the intensities on the two sides of the contour *C*, respectively.

This energy can be expressed by a level set formulation, and then the image segmentation problem can be converted to minimizing the energy functional *F*(*φ*, *f*_1_, *f*_2_) by solving the level set evolution equation as follows:

2 $$\frac{\partial \phi }{\partial t} = - \delta_{\varepsilon } (\phi )(\lambda_{1} e_{1} - \lambda_{2} e_{2} ) + \nu \delta_{\varepsilon } (\phi )div\left( {\frac{\nabla \phi }{\nabla \phi }} \right) + \mu \left( {\nabla^{2} \phi - div\left( {\frac{\nabla \phi }{|\nabla \phi |}} \right)} \right)$$

with

$$e_{1} = \int {K_{\sigma } (y - x)\left| {I(x) - f_{1} (y)} \right|^{2} dy} \,\,\,\,\,\,\,\,\,e_{ 2} = \int K_{\sigma } \left( {y - x} \right)\left| {I\left( x \right) - f_{ 2} \left( y \right)} \right|^{ 2} dy$$

$$f_{1} = \frac{{K_{\sigma } * \left( {H_{\varepsilon } (\phi )I(x)} \right)}}{{K_{\sigma } * H_{\varepsilon } (\phi )}}\,\,\,\,\,\,\,\,\,\,\,\,f_{2} = \frac{{K_{\sigma } * \left( {(1 - H_{\varepsilon } (\phi )} \right)I(x))}}{{K_{\sigma } * \left( {1 - H_{\varepsilon } (\phi )} \right)}}$$

Due to the localization property of the kernel function, local intensity information is extracted to guide the evolution of the active contour, which thereby enables the RSF model to achieve promising results. However, because of many local minimums of the energy functional which are introduced by such localization property, the segmentation results are sensitive to contour initialization.

### The local robust statistics (LRS) model

Gao [70] proposed a local robust statistics based conformal metric and the conformal area driven multiple active contour framework, to simultaneously segment multiple objects from 3D medical imagery. Let $I:\varOmega \to \Re$ where $\varOmega \subset \Re^{d}$ and $d \in \left\{ { 2, 3} \right\}$ be the image to be segmented, $C_{i} \subset \varOmega$ be the family of evolving closed contour. The variable *x* in *f*(*x*) is a point in *Ω*. Without interactions among contour, they proposed the following energy functional:

3 $$E_{i} (C_{i} ): = (1 - \lambda )\int_{{\begin{array}{*{20}c} x & {in} & {C_{i} } \\ \end{array} }} {\left( {p^{c} - p_{i} \left( {f(x)} \right)} \right)dx} + \lambda \int_{{C_{i} }} {dA}$$

where in the first term the seed groups are characterized by the probability density function *p*_*i*_(*f*(*x*)) of the robust statistics feature vectors *f*(*x*) whose variance can be adjusted according to the intensity inhomogeneity of the target and the second term is the surface area. Moreover, the *p*^*c*^ is the cut-off probability density used to prevent the contour leakage [71] and is fixed at 0.1 as suggested there. The smoothness factor *λ* is nonnegative constant.

With the interaction among curves, the energy functional in Eq. 3 is updated as

4 $$E_{i} (C_{i} ): = (1 - \lambda )\int_{{\begin{array}{*{20}c} x & {in} & {C_{i} } \\ \end{array} }} {\left( {p^{c} - p_{i} \left( {f(x)} \right)} \right)dx} + \lambda \int_{{C_{i} }} {dA} + \sum\limits_{j \ne i} {\int_{{\begin{array}{*{20}c} x & {in} & {C_{j} } \\ \end{array} }} {e^{{ - |p_{i} - p_{j} |}} \left( {p^{c} - p_{j} \left( {f(x)} \right)} \right)dx} }$$

where the third term estimates the speeds of the curves except *C*_*i*_ and the exponential term in the third term controls the influence range of the speed. By minimizing the energy functional, the contours evolve towards the objects boundaries.

**Fig. 1:**
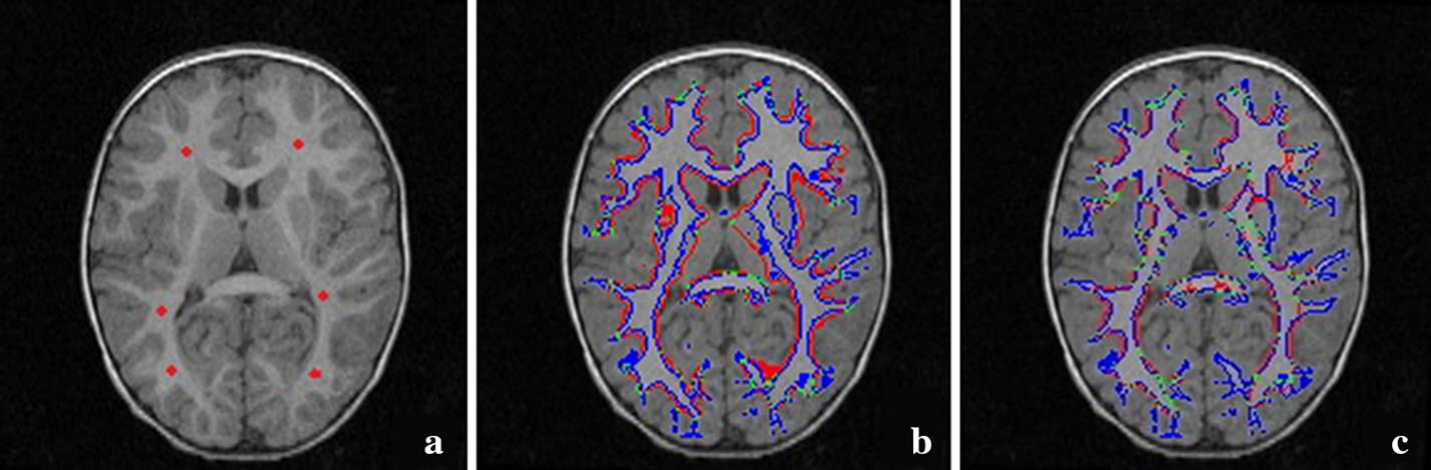
Results of the LRS model. **a** An axial slice of the original volumetric image and the initial seeds (shown in *red*). **b**, **c** The segmentation results with lower intensity inhomogeneity hypothesis and higher intensity hypothesis in parameter setting, respectively. The *red contour* is generated by the LRS model, while the *blue* *contour* represents expert manual segmentation, and the green contour is where the algorithm ones coincide with the manual ones

Such evolution is a curve expansion scheme which tries to maximize the area the surface encloses. Because the interactions between the contours are incorporated into the evolution, the contour leakage is effectively reduced. Whereas, an unsophisticated *p*^*c*^ term in Eq. 4 may stop the contour evolving before the interactions happening, if the intensities in both sides of the curves are inhomogeneous. Moreover, without taking similarity of adjacent points into account, the segmentation results of the LRS model for images with inhomogeneity are not sufficiently satisfying. For example, Fig. 1 shows the initial seeds and the segmentation results for a brain volumetric MR image with intensity inhomogeneity and weak object boundary [72]. It can be seen from Fig. 1b that a lower intensity inhomogeneity hypothesis in parameter setting causes the contour leakage, while some detailed regions are still under-segmented. On the other hand, if a higher intensity inhomogeneity is hypothesized, the under-segmentation is severe, as shown in Fig. 1c.

In this paper, we propose a novel volume-scalable ACM via PDF based description of local robust statistics for volumetric medical image segmentation. Specifically, we first define a feature vector with volume-scalable robust statistics in order to make better use of volumetric image information, which are presented by the initial seeds and the local volumes on the two sides of the evolving contour, rather than just using image intensity. A kernel function controls the volume-scalability of the robust statistics with a scale parameter, which allows the use of local statistics information around the center voxels at a flexible scale. We then define a volume-scalable fitting energy functional in terms of two fitting functions that derived from feature vectors afore mentioned. This energy is then incorporated into a variational level set formulation with a level set regularization term and a smooth term. In the associated curve evolution, the motion of the active contour is driven by the two fitting functions, induced by the robust statistics information in local volumes at a certain scale. As a result, the proposed model can be used to segment volumetric medical images with intensity inhomogeneity as well as noisy weak object boundary.

## Methods

Our approach for the segmentation of volumetric medical images is based on a new energy functional. In this section, we first describe the original robust statistics fitting energy formulation of the functional in local volumes at multi-scales. Then, we introduce a reformulation as variational level set model.

### Volume-scalable robust statistics feature vector learning

Denote the vector valued image to be segmented as $I:\varOmega \to \Re^{d}$ where $\varOmega \subset \Re^{ 3}$ is the image domain, and *d* ≥ 1 is the dimension of the vector *I*(*x*). Let $L:\varOmega \to \left\{ {0, 1} \right\}$ be a label map in the image domain *Ω*, which separates *Ω* into the target object volume: $\varOmega_{ 1} = \{ x \subset \varOmega :L\left( x \right) = 1\}$ and the background volume: $\varOmega_{ 2} = \{ x \subset \varOmega :L\left( x \right) = 0\}$. In particular, $\varOmega_{Seeds\_1}$ and $\varOmega_{Seeds\_2}$ indicate the user provided seeded target object volume and seeded background volume, respectively.

In images with complex conditions, general information about the target/background given by the label map in 2D, such as image intensity and location of the target, are not descriptive enough. For fully utilizing the information provided by the initial label map in 3D, not only the locations of the seeds, but also some sample voxels contained in seed volumes are taken into account in this work. Hence, more information can be extracted at each voxel and a feature image $f:\varOmega \to \Re^{{D_{f} }}$ is formed. Then, images are segmented with the feature image assisted. In this work, we choose local robust statistics [73] to construct the feature vectors for their insensitivity to image noise and computational efficiency.

Numerically, in computing the robust statistics in local volumes at a controllable scale and assigning different weights to the data for voexls according to their distance to the central voxel, we define the weighting neighborhood using a non-negative kernel function *K* such that *K*(*u*) ≤ *K*(*v*) for |*u*| > |*v*| and $\smallint K\left( x \right)dx = 1$

There are various choices for the kernel function. In this work, we use the Gaussian kernel

5 $$K_{\sigma } (u) = \frac{1}{{(2\pi )^{n/2} \sigma^{n} }}e^{{{{ - |u|^{2} } \mathord{\left/ {\vphantom {{ - |u|^{2} } {2\sigma^{2} }}} \right. \kern-0pt} {2\sigma^{2} }}}}$$

with a scale parameter *σ* > 0.

Then, within the kernel controlled neighborhood *B*(*x*) ⊂ *Ω* of voxel *x*, we define the feature vector $f(x) \in \Re^{{D_{f} }}$ for each voxel *x*$\in$*Ω* by combining several volume-scalable robust statistics. More explicitly, we denote

6 $$VSMEAN(x): = \frac{{K_{\sigma } (x) * I(x)}}{{K_{\sigma } (x) * 1}}$$

as the volume-scalable intensity mean value within *B*(*x*). In addition, for bypassing the influence of outliers when calculating the local intensity range, the distance between the first and third kernel function weighted quartiles, namely the volume-scalable inter-quartile range *VSIQR*(*x*), is calculated as the second feature. Furthermore, the intensity variance is a good character for the local volume but again it is sensitive to outliers. To improve the robustness, the weighted intensity variance is chosen to be the third feature and is calculated as

7 $$WIV(x): = \left( {\frac{{K_{\sigma } (x) * \left( {I(x) - VSMEAN(x)} \right)^{2} }}{{K_{\sigma } (x) * 1}}} \right)^{{\frac{1}{2}}}$$

Consequently, the feature vector *f*(*x*) is defined as

8 $$f(x) = (VSMEAN(x),VSIQR(x),WIV(x))^{T} \in \Re^{3}$$

It is necessary to elaborate on the meaning of the feature vector in the following. First, *f*(*x*) is a weighted statistics of the voxels *y* in the neighborhood of the center voxel *x*, with $K_{\sigma } \left( {x - y} \right)$ as the weight assigned to each voxel *y* via convolution operation. Due to the localization property of the kernel function, the voxels that are close to the center voxel and give more contribution to the robust statistics are assigned high weight. On the contrary, the voxels that are far away from the center voxel are assigned low weight. Therefore, the feature vector *f*(*x*) is dominated by the voxels *y* in a neighborhood of *x*. Second, the feature vector is volume-scalable in the following sense. The feature vector approximate the image character in a volume centered at the voxel x, whose size can be controlled by the kernel function. In particular, the Gaussian kernel with a large *σ* specify a large neighborhood of the voxel *x*, while the Gaussian kernel with a small *σ* specify a small volume centered at *x*. In this sense, we say that the feature vector is volume-scalable.

### Voxel characterization using the PDFs of the feature vectors

With the volume-scalable feature vectors defined in Eq. 8, each voxel *x* can be characterized by combining the PDFs of the feature vectors derived in the seeded volumes with that derived in a neighborhood around voxel *x*. The characterization of voxel *x* is then described as follows:

9 $$P_{i} (x) = (1 - \omega )\frac{1}{{|\varOmega_{Seeds\_i} |}}\sum\limits_{{z \in \varOmega_{Seeds\_i} }} {p\left( {f(x) - f(z)} \right)} + \omega \int_{{\varOmega_{i} }} {K_{\eta } (x - y)p\left( {\mu_{i} (x) - f(y)} \right)dy} ,\begin{array}{*{20}c} {} & {i = 1,2} \\ \end{array}$$

where in the first term the *z* is the seed voxel belongs to seed volume *Ω*_*Seeds*_*i*_ and the second term is a weighted average of the probability distribution *p* in a neighborhood of voxel x, whose size is controlled by the scale parameter *η* of the kernel function *K*_*η*_ given by Eq. 5. Moreover, the *μ*_*i*_ in the probability density approximate image characters in local volume *Ω*_*i*_ which will be formulated in Section “Energy minimization”. Note that the choices to model the probability distribution *p* in Eq. 9 are flexible. In this work, we use the Gaussian distribution, whose variance can be adjusted according to the *WIV* of the voxels which were used to characterize the voxel *x*.

**Fig. 2:**
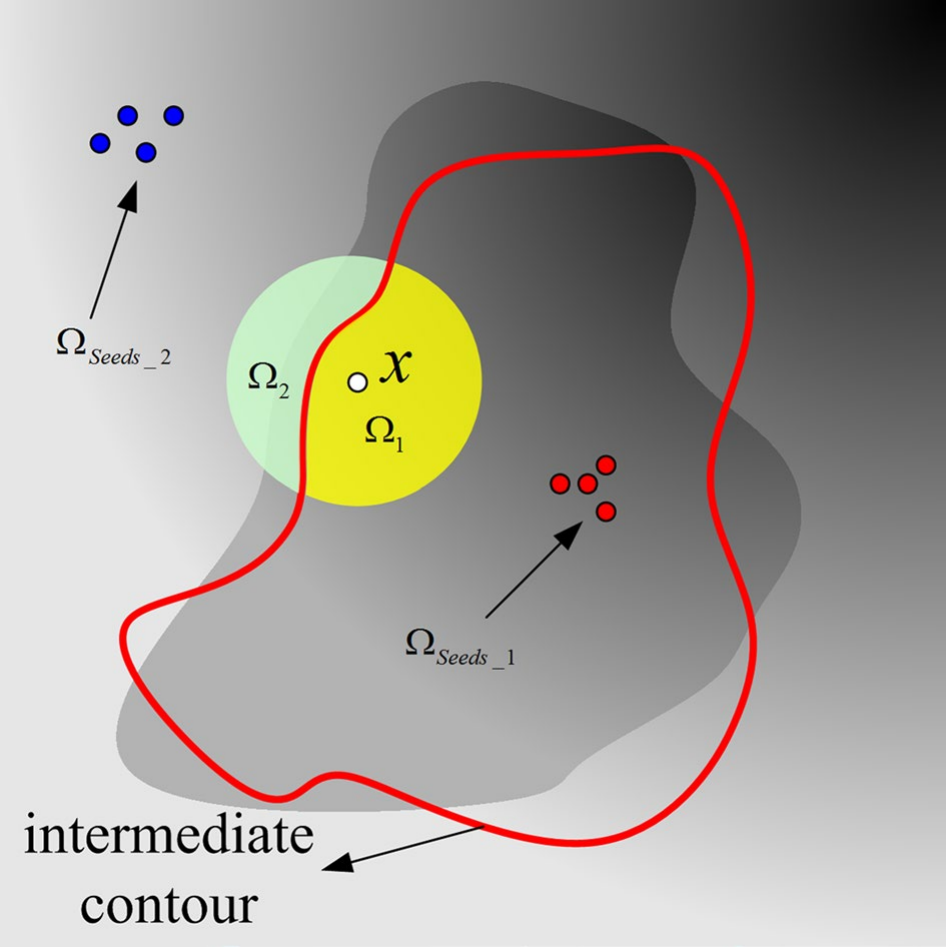
Characterization of a center voxel *x*. Voxel *x* is characterized by the voxels in a neighborhood around voxel *x* and in the seeded regions, respectively

The *ω* in Eq. 9 is a positive constant (0 ≤ *ω* ≤ 1), and it balance the importance of user selected seeds and the local volumes. This can be illustrated by a 2D example shown in Fig. 2. The initial seeded foreground region *Ω*_*Seeds*_1_ and seeded background region *Ω*_*Seeds*_2_ are plotted in red and blue points, respectively. The yellow and green regions represent the intermediate local foreground region *Ω*_1_ and local background region *Ω*_2_, respectively. It can be seen that when a specific object is desired, the voxel *x* should be characterized mainly by the seeded region and the parameter value *ω* should be chosen small enough. The selection of the parameter *ω* is discussed in “Discussion” section.

### Definition of volume-scalable robust statistics fitting energy for single voxel

With the user provided label map *L*, for a given voxel $x \in \varOmega$, we define the following volume-scalable robust statistics fitting (VSRF) energy:

10 $$\varepsilon_{x}^{VSRF} (L,P_{1} ,P_{2} ) = \sum\limits_{i = 1}^{2} {\lambda_{i} \int_{{\varOmega_{i} }} {K_{\eta } (x - y)P_{i} (y)dy} }$$

where *λ*_1_ and *λ*_2_ are positive constants, *P*_1_ and *P*_2_ are two values defined in Eq. 9 that characterize image voxels with seeded volumes and neighbor volumes. *K*_*η*_ is the kernel function given by Eq. 5, which control the size of a local volume centered at the voxel *x*. Then, the statistic character of voxels in the neighbor volume of voxel *x* are effectively involved in the above fitting energy. In this sense, the robust statistics fitting energy is also volume scalable.

Contrasting to the ordinary region based schemes where the fitting energy is minimized, here we try to maximize the fitting energy *ɛ*_*x*_^*VSRF*^ in Eq. 10. Essentially, if in certain volume the label *L* is exactly separating the object from the background and the fitting values *P*_1_ and *P*_2_ optimally approximate the local robust statistics character of voxels with different labels, the value of fitting energy *ɛ*_*x*_^*VSRF*^ is big. Therefore, partial object boundary can be achieved. However, in the following discussion the energy term will be integrated with regularization terms which need to be minimized. For unification, the maximization needs to be converted to the minimization. There are various approaches to perform the conversion. In this work, the maximization of the energy in Eq. 10 is extended by simply adding a negative sign, obtaining a new fitting energy given by

11 $$\varepsilon_{x}^{VSRF} (L,P_{1} ,P_{2} ) = - \sum\limits_{i = 1}^{2} {\lambda_{i} \int_{{\varOmega_{i} }} {K_{\eta } (x - y)P_{i} (y)dy} } .$$

### Definition of energy functional for entire volumetric data

Now, we define the following energy functional:

12 $$\varepsilon^{VSRF} (L,P_{1} ,P_{2} ) = \int_{\varOmega } {\varepsilon_{x}^{VSRF} (L,P_{1} ,P_{2} )dx}$$

where the fitting energy *ɛ*_*x*_^*VSRF*^ is integrated over all the center voxels *x* in the 3D image domain *Ω*. By minimizing the integral, we can obtain the entire object boundary. We will give a level set formulation of the energy functional in the next subsection.

### Volume-scalable level set formulation

Let *φ* be the level set function and *H*(·) be the Heaviside function, and then the fitting energy *ɛ*_*x*_^*VSRF*^ (*L*, *P*_1_, *P*_2_) can be expressed as

13 $$\begin{aligned} \varepsilon_{x}^{VSRF} (\phi ,P_{1} ,P_{2} ) &= - \lambda_{1} \int {K_{\eta } (x - y)P_{1} (y)H\left( {\phi (y)} \right)dy} \\ &\quad- \lambda_{2} \int {K_{\eta } (x - y)P_{2} (y)\left( {1 - H\left( {\phi (y)} \right)} \right)dy} \\ \end{aligned}$$

Thus, we define the following energy functional:

14 $$\begin{aligned} \varepsilon^{VSRF} (\phi ,P_{1} ,P_{2} ) &= - \lambda_{1} \int {\left( {\int {K_{\eta } (x - y)P_{1} (y)H\left( {\phi (y)} \right)dy} } \right)dx} \\ &\quad - \lambda_{2} \int {\left( {\int {K_{\eta } (x - y)P_{2} (y)\left( {1 - H\left( {\phi (y)} \right)} \right)dy} } \right)dx} \\ \end{aligned}$$

As in most level set methods [11, 74], we introduce a level set regularization term and a smoothness term in our variational level set formulation. The level set regularization term can be defined as

15 $${\rm P}(\phi ) = \int {\frac{1}{2}\left( {\left| {\nabla \phi (x)} \right| - 1} \right)^{2} dx}$$

and the smoothness term can be defined as

16 $$L(\phi ) = \int_{\varOmega } {\left| {\nabla H\left( {\phi (x)} \right)} \right|dx}$$

Then, we define the entire energy functional

17 $$F(\phi ,P_{1} ,P_{2} ) = \varepsilon^{VSRF} (\phi ,P_{1} ,P_{2} ) + \nu L(\phi ) + \mu P(\phi )$$

where *ν* and *μ* are positive constants.

### Contour evolution with level set energy functional minimization

Keeping *φ* fixed and minimizing the energy functional *F*(*φ*, *P*_1_, *P*_2_) in Eq. 17 with respect to the functions *P*_1_ and *P*_2_, we deduce the following optimal expressions for the functions *P*_1_ and *P*_2_ that minimize *F*(*φ*, *P*_1_, *P*_2_):

18 $$\begin{aligned} P_{1} (x) &= (1 - \omega )\frac{1}{{|\varOmega_{Seeds\_1} |}}\sum\limits_{{z \in \varOmega_{Seeds\_1} }} {p\left( {f(x) - f(z)} \right)} + \omega \int {K_{\eta } (x - y)p\left( {\mu_{1} (x) - f(y))H(\phi (y)} \right)dy} , \\ P_{2} (x) &= (1 - \omega )\frac{1}{{|\varOmega_{Seeds\_2} |}}\sum\limits_{{z \in \varOmega_{Seeds\_2} }} {p\left( {f(x) - f(z)} \right)} + \omega \int {K_{\eta } (x - y)p\left( {\mu_{2} (x) - f(y)} \right)\left( {1 - H\left( {\phi (y)} \right)} \right)dy} \hfill \\ \end{aligned}$$

With

19 $$\begin{aligned} \mu_{1} (x) &= \frac{{\int {K_{\eta } (x - y)f(y)H\left( {\phi (y)} \right)dy} }}{{\int {K_{\eta } (x - y)H\left( {\phi (y)} \right)dy} }}, \hfill \\ \mu_{2} (x)& = \frac{{\int {K_{\eta } (x - y)f(y)\left( {1 - H\left( {\phi (y)} \right)} \right)dy} }}{{\int {K_{\eta } (x - y)\left( {1 - H\left( {\phi (y)} \right)} \right)dy} }} \hfill \\ \end{aligned}$$

Keeping *P*_1_ and *P*_2_ fixed, we minimize the energy functional *F*(*φ*, *P*_1_, *P*_2_) in Eq. 17 with respect to *φ* using first variation of *F* by solving the gradient descent flow of *φ* as follows:

20 $$\frac{\partial \phi }{\partial t} = \delta (\phi )(\lambda_{1} e_{1} - \lambda_{2} e_{2} ) + \nu \delta (\phi )\,div\left( {\frac{\nabla \phi }{|\nabla \phi |}} \right) + \mu \left( {\nabla^{2} \phi - div\left( {\frac{\nabla \phi }{|\nabla \phi |}} \right)} \right)$$

where *δ* is the Dirac delta function, and *e*_1_ and *e*_2_ are the functions

21 $$e_{i} (x) = \int {K_{\eta } (x - y)P_{i} (y)dy} ,\begin{array}{*{20}c} {} & {i = 1,2} \\ \end{array}$$

in which *P*_1_ and *P*_2_ are given by Eq. 18.

In the proposed method, the segmentation problem can be solved by evolving the level set equation Eq. 20. The first term in Eq. 20 makes sure that the active contour can evolve toward object boundary. The second and third term maintain the smoothness of the zero level contour and the regularity of the level set function, respectively.

## Results

All the spatial partial derivatives of *φ* can be discretized with a finite differences scheme as developed in [75]. The temporal derivative is discretized as a forward difference with a semi-implicit Gauss–Seidel method [76]. In order to learn the object features and start contour evolution, the initial label map is needed, and this can be done with ease by drawing some seeds/strokes in some slices of the volumetric image. Accordingly, the level set function *ϕ* can be simply initialized as a signed distance function. Then, the level set function *ϕ* is updated iteratively after the update of the fitting functions *P*_1_ and *P*_2_ at every time step. The volume rendering was implemented using the “Model Maker” module in 3D Slicer [77].

The proposed method has been extensively tested with synthetic and real volumetric medical imagery segmentation datasets. Unless otherwise specified, we set the following default values for the parameters in our method:

$$\sigma = 0.5,\,\eta = 3.0,\,\,\lambda_{1} = 1.0,\,\,\lambda_{2} = 2.0,\,\,timeStep\,\,\,\begin{array}{*{20}c} {} & {\varDelta t = 0.1,\,\,\omega = 0.4,\,\,\mu = 1.0,} \\ \end{array} \,\,{\text{and}}\begin{array}{*{20}c} {} & {\nu = 0}. \\ \end{array}$$

The influence of different key parameters on the segmentation results of the proposed method will be discussed in “Discussion” section.

**Fig. 3:**
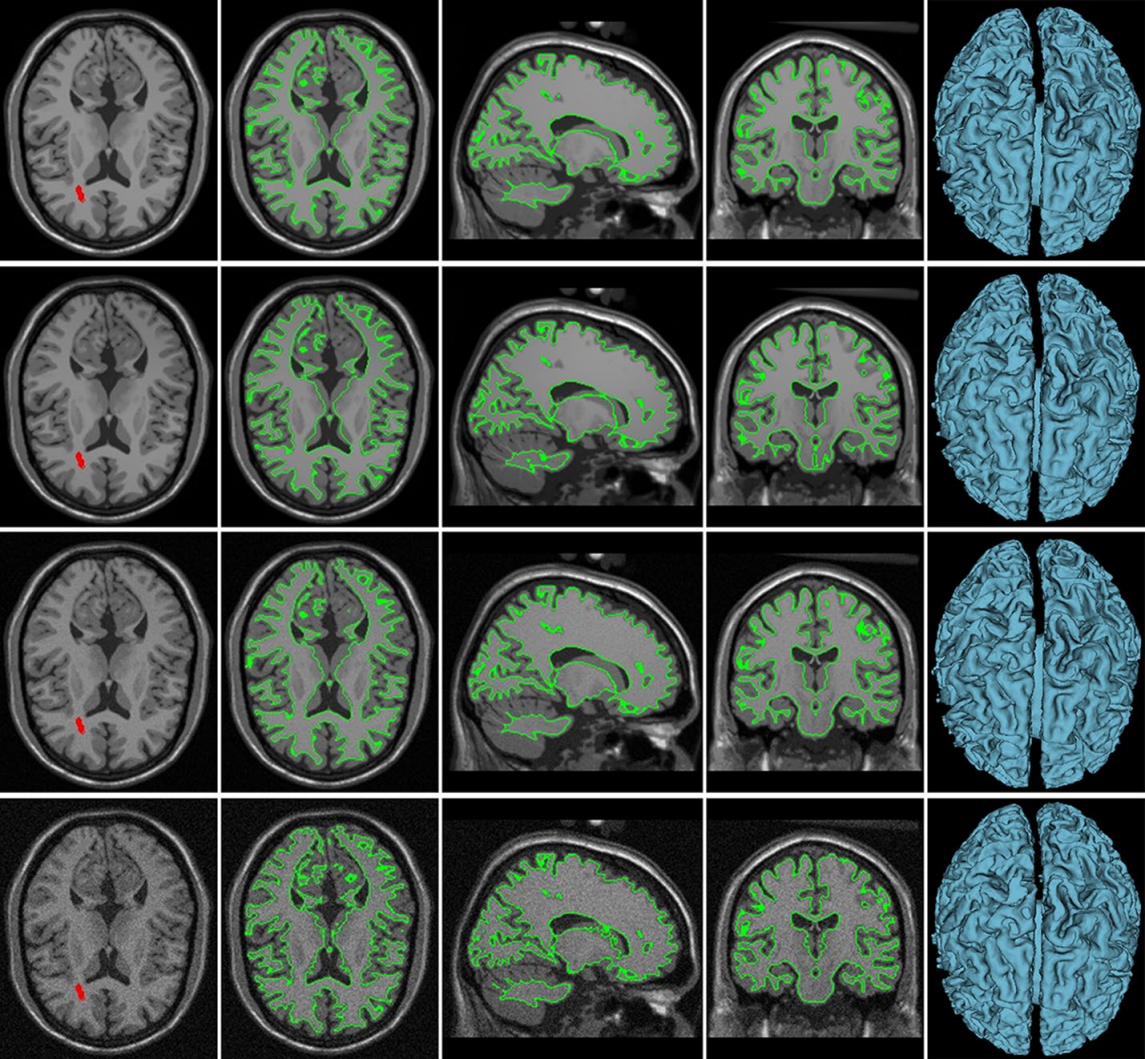
Performances of our method for synthetic images with different degrees of intensity inhomogeneity and noise. *Column 1* one axial slice of the original brain volumetric MR images and the initial seeded labels (*red strokes*). *Columns 2, 3 and 4* segmentation results (*green contours*) in axial, sagittal, and coronal views. *Column 5* the 3D surface model of the segmentation results

We first show the results for four synthetic brain volumetric MR images with slice thickness 1 mm and image size 180 × 180 × 216, which are downloaded from BrainWeb [78–81], in Fig. 3. These images have the same WM but different degrees of intensity inhomogeneity and different levels of noise. We draw the initial seeds on the images in the first column to learn the object features and start contour evolution. Although we only show some strokes with free locations in one slice of the volumetric images, indeed, the seeds can be drawn anywhere inside the volumetric images. The details about the initial contours will be shown in Fig. 7. The final contours in three standard views are shown in the middle three columns and the last column gives 3D surface models of the segmented objects.

The first row in Fig. 3 shows the segmentation result for the image with 20 % degree of intensity non-uniformity (INU). It can be seen that our method can handle intensity inhomogeneity well and successfully extracts the object boundaries. The second row shows the segmentation result of the image with 40 % degree of INU. As can be observed, despite the severe intensity inhomogeneity in the image, our method is able to produce satisfactory segmentation result.

In order to test the robustness of our method to noise, we generated the third and forth rows in Fig. 3 by adding zero mean Gaussian noise into the 20 % INU contaminated image in the first row with standard deviation (STD) of 10 and 30, respectively. Reinforced by the robust statistics features, our method is robust to noise contamination, which is confirmed by the segmentation results shown in the third and forth rows. In the third row, it can be seen that although the STD of the Gaussian noise is 10 which is high relative to the image contrast ranging from 0 to 200, the algorithm can still segment the image very well. In the forth row, the STD of added noise increases to 30, and some part of the object boundary is blurred heavily by the strong noise. In this case, although the segmentation result is not as good as before, the algorithm still captures the WM correctly, which demonstrates the advantage of our method in terms of the robustness to the noise.

By visual inspection, the proposed method achieves desirable performances for these synthetic images with various INU degree and noise level, which is also confirmed by the HD values as provided in Table 1.

**Table 1:** The HD values of segmenting the WM, under various conditions, comparing with the ground truth

INU degree and noise STD	20 % INU and 0 STD	40 % INU and 0 STD	20 % INU and 10 STD	20 % INU and 30 STD
HD (mm)	1.76	7.65	3.9	10.06

**Fig. 4:**
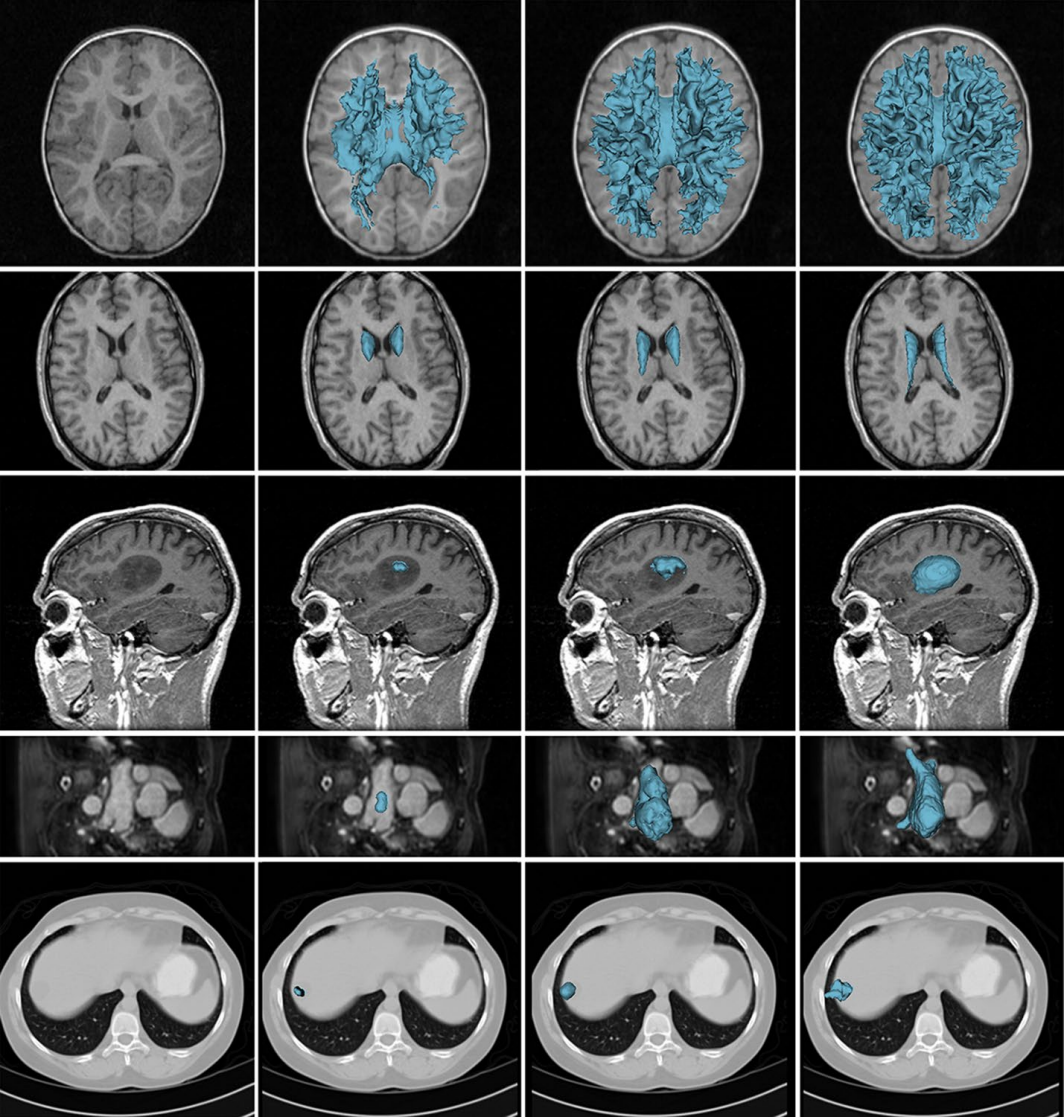
Segmentation results of our method for real volumetric medical images. *Column 1* original images; *columns 2 and 3* the intermediate 3D surface models; *column 4* the 3D surface model of the final segmentation results

Intensity inhomogeneity and noisy weak object boundary often occur in real medical images, which render it a nontrivial task to segment the target from the background accurately. Figure 4 shows the results of the proposed method for five typical volumetric medical images with complex image conditions. In particular, the brain MR image in the first row has been used in Fig. 1, by which we have demonstrated that the LRS model fails to segment the images due to the unsophisticated parameter setting and the intensity inhomogeneity.

The first row shows the result for a brain MR image which is corrupted severely by intensity inhomogeneity. In this image, some parts of the WM have even lower intensities than parts of the gray matter (GM) have. The second row shows the result for a MR image of caudate nucleus [82]. Due to the poor contrast with the surrounding tissues, it is difficult to extract the caudate nucleus from the background. The third row shows the result for a MR image of a brain with meningioma [83]. As can be seen in this image, parts of the meningioma boundaries are blurred. The forth and the bottom rows show the results for a MR image of left atrium [84] and a CT image of liver tumors [85], respectively. Both of the images are contaminated with weak object boundaries due to partial volume effect and low contrast, respectively. Our method has successfully extracted the object boundaries in these challenging images, as shown in the last column of Fig. 4. The advantage of the proposed method in terms of accuracy is also confirmed by the average DSC values for WM, caudate nucleus, left atrium and liver tumors on all subjects within each above-mentioned dataset. The meningioma is not included in calculating the average DSC value because there is only one subject in this dataset. Specifically, the average DSC value are 0.9246 ± 0.0068 (WM), 0.8725 ± 0.0374 (caudate nucleus), 0.9205 ± 0.0146 (left atrium) and 0.9043 ± 0.0131 (liver tumors), respectively.

**Fig. 5:**
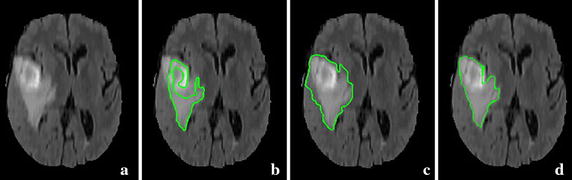
The original axial slices are overlaid with the segmentation results of three different segmentation method. **a** The axial slice from the original volumetric data. **b** Result of the RSF model. **c** Result of the SBGFRLS model. **d** Result of our method

Figure 5 shows the brain tumor segmentation of a real volumetric brain MR image with obvious intensity inhomogeneity and weak object boundary. Brain tumor image data used in this work were obtained from the MICCAI 2012 Challenge on Multimodal Brain Tumor Segmentation organized by B. Menze, A. Jakab, S. Bauer, M. Reyes, M. Prastawa, and K. Van Leemput. The challenge database contains fully anonymized images from the following institutions: ETH Zurich, University of Bern, University of Debrecen, and University of Utah [86].

For this image from single modality, we aim at segmenting the brain tumor and the edema volumes integrally. From left image to the right one are the axial slice extracted from the original volumetric MR image, the results obtained by two well-known 2D methods: the RSF model and the SBGFRLS model [28], and by our 3D segmentation method, respectively. For this and the following brain tumor images, we use the following parameters in our model:

$$\sigma = 0.5,\,\,\eta = 3.0,\,\,\lambda_{1} = \lambda_{2} = 1.0,\,\,timeStep\,\,\begin{array}{*{20}c} {} & {\varDelta t = 0.1,\,\,\omega = 0.1,\,\,\mu = 1.0,} \\ \end{array} \,\,{\text{and}}\begin{array}{*{20}c} {} & {\nu = 0}. \\ \end{array}$$

Note that we choose a smaller value *ω* for these images to cope with the severe intensity inhomogeneity by strengthening the influence of the intensity in the desired target (the tumor region), as explained in “Discussion” section. In this and the following experiments, we choose smaller *λ*_2_ and *ν* than in the previous experiments to further encourage the expansion of the contour to the inhomogeneous hybrid volumetric region. As can be seen from Fig. 5, because the intensity of the hybrid lesion region with both brain tumor and edema are very inhomogeneous and some intensities of the edema regions in the right and middle parts are very similar to those of the adjacent non-lesion regions, the results (shown as green contours) obtained by the RSF model and the SBGFRLS model are less accurate: part of the tumor is incorrectly identified as the non-lesion region, while parts of non-lesion regions are included into the result. By contrast, our method segments the lesions more accurately, which demonstrate the advantage of our method over these 2D segmentation methods.

**Fig. 6:**
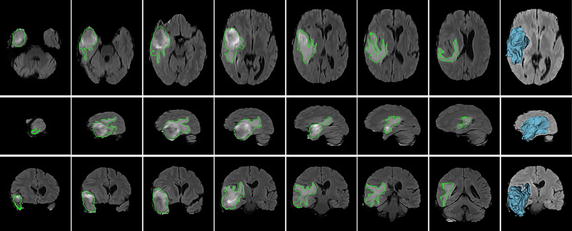
Multiple slices of the 3D segmentation results in Fig. 5d. The final contours (shown as *green contours* in the *1–7 columns*) and the 3D surface models (shown as *blue volumes* in the *last column*) overlaid on the corresponding axial slices (in the *first row*), the sagittal slices (in the *second row*) and the coronal slices (in the *last row*), respectively

To demonstrate the advantage of our method in terms of accuracy more clearly, we show multiple slices in three standard views and the final segmentation result obtained by our method in Fig. 6. It can be clearly seen that our method correctly recovers the boundaries of the brain tumor in the volumetric MR image.

**Fig. 7:**
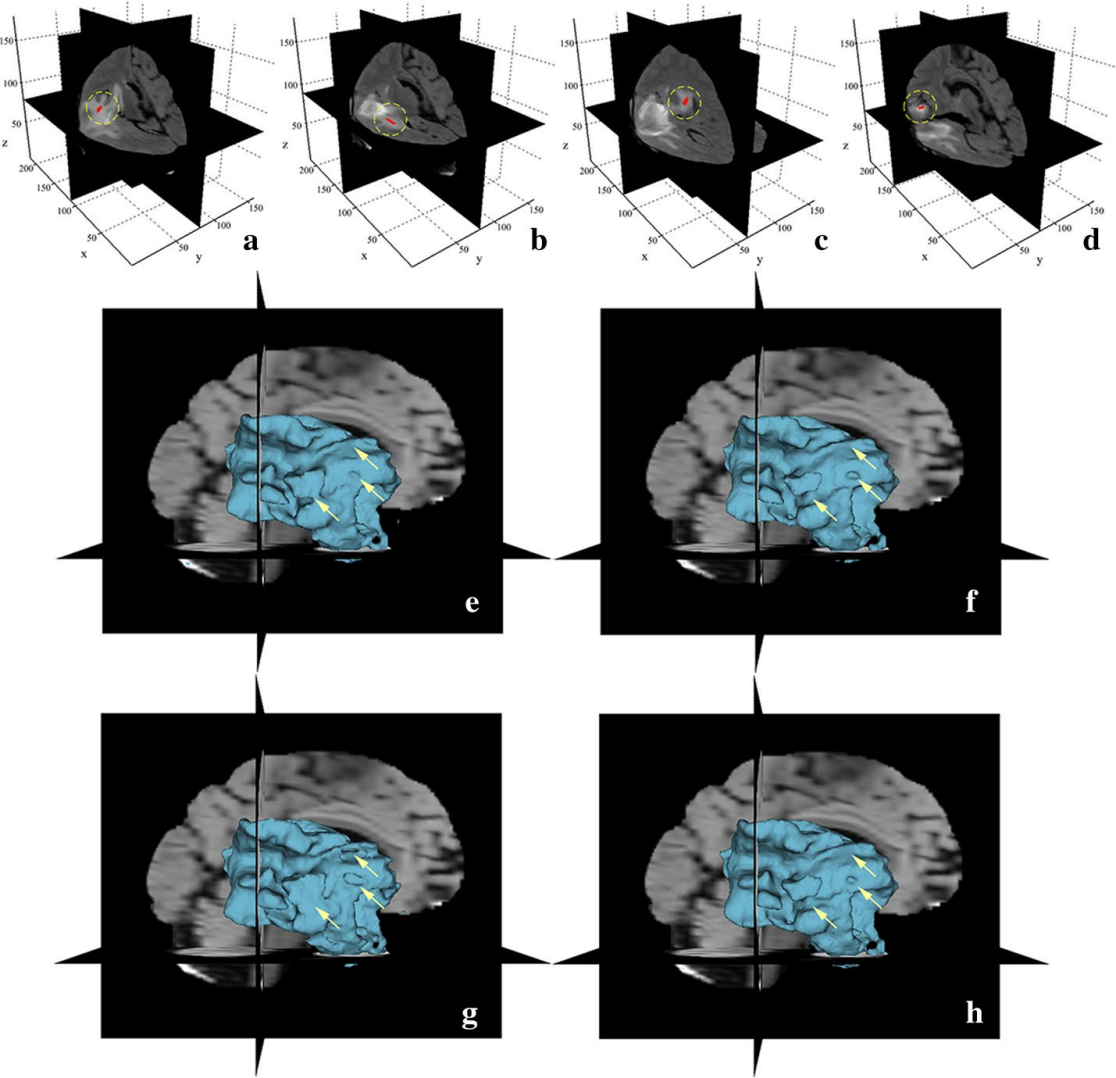
Facilitation and robustness of our method to contour initializations. **a**–**d** Different initial seeded labels (*red strokes* indicated by *dashed yellow circles*) are drawn inside the same target. **e**–**h** The corresponding 3D surface models (*blue volumes*) and some main differences of the resulting segmentations (indicated by *arrows*)

We evaluate the performance of our method with 10 different initializations of the contour for the same volumetric image which has been used in Fig. 6. The orthogonal slice views in Fig. 7a–d show four of the 10 initial seeded contours. The corresponding 3D surface models overlayed on the three orthogonal slices, are shown in Fig. 7e–f. Note that in these four different initializations, the initial contours are simplified as seeded labels, which are different from the closed initial contours in traditional ACMs and, therefore, facilitate user interaction. Moreover, instead of roughly drawing the seeds around the center region of the target as proposed in [70], we draw the initial seeded labels in random position in our method as long as they are inside the target. It can be seen from Fig. 7 that despite the great difference of these initializations, the corresponding segmentation results are quite consistent with each other. The boundaries of the objects of interest (the lesion regions) are accurately captured for these different initializations. The average DSC value for the 10 segmentation results with different initializations is 0.9216 ± 0.0037, which again demonstrates the advantage of the proposed method in terms of robustness to contour initialization.

### Validation and method comparison

Previous experimental results for the RSF model and the SBGFRLS model shown in Fig. 5 and those of the proposed method shown in Figs. 3, 4, 5, 6 and 7 have demonstrated the advantages of the proposed method over these three models. We now quantitatively evaluate and compare the performances of the proposed method and the well-known 3D segmentation softwares ITK-SNAP [87], Seg3D [88] and 3D Slicer [77].

**Fig. 8:**
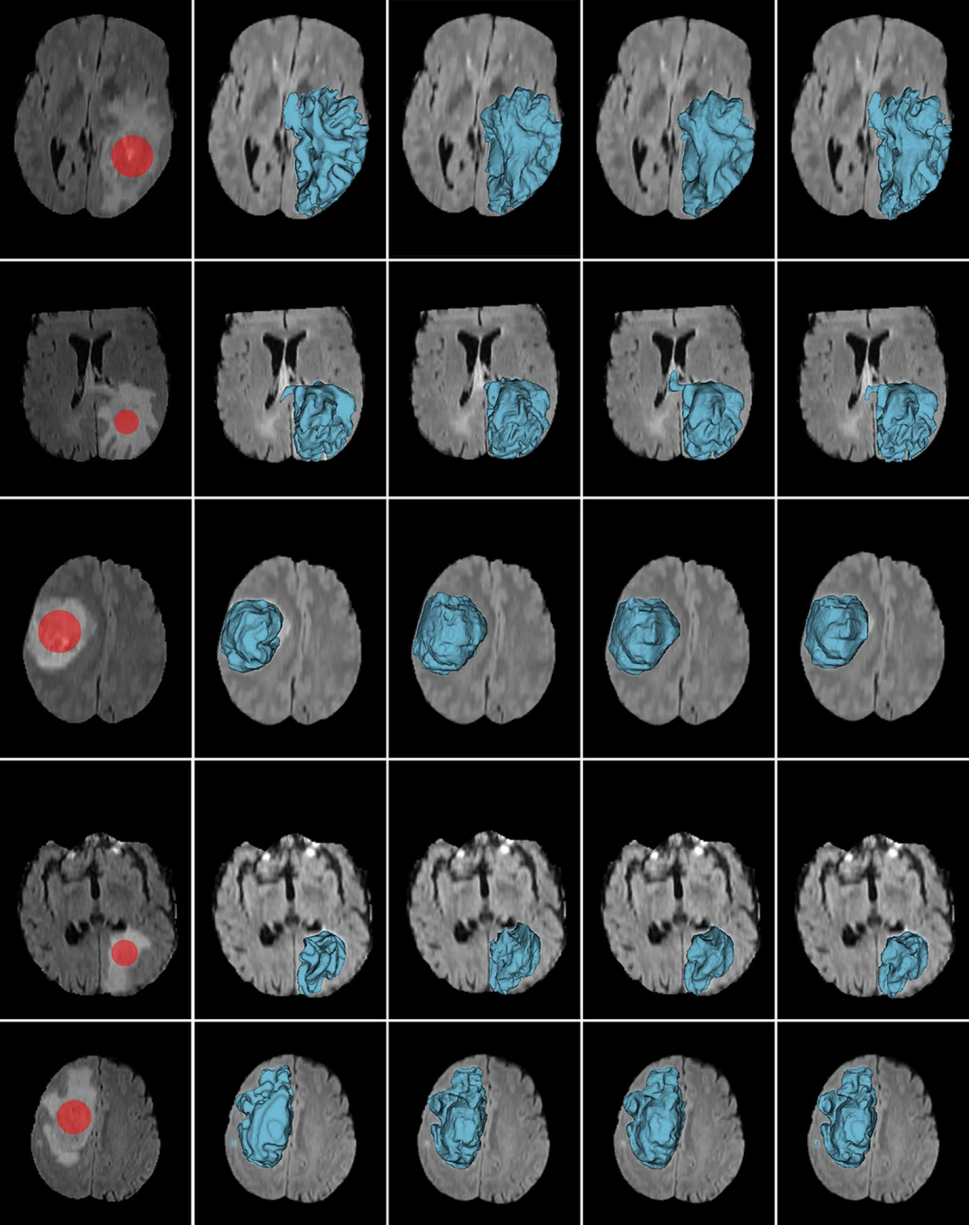
Comparison of our method with ITK-SNAP and Seg3D on five real brain tumor volumetric MR images. *Column 1* the initial labels (*red bubbles*) and slices of the original volumetric images with complex image conditions; *column 2* ground truth; *column 3* results of ITK-SNAP for five images with region competition force *α* and the smoothing force *β* represented as a pair (*α*, *β*) = (0.65, 0.35), (0.65, 0.35), (0.55, 0.25), (0.5, 0.03), and (0.7, 0.25) in the order from *left* to *right*; *column 4* results of Seg3D with threshold range *TR*, curvature weight *CW*, and propagation weight *PW* represented as a triple (*TR*, *CW*, *PW*) = (2,5, 0.1), (3.8, 7, 0.1), (2.55, 3, 0.1), (2.3, 3, 0.1), and (2.1, 3, 0.1) in the order from *left* to *right*; *column 5* results of the proposed method

We first show the results of comparison with ITK-SNAP and Seg3D. ITK-SNAP and Seg3D are two very nice open-source softwares providing optional algorithms for 3D image segmentation, both which can achieve excellent results by incorporating the algorithms with expert manual refinements in the friendly software environment. In this work, we employ the region competition based active contour algorithms, which are embedded in these softwares, for comparison without further manual segmentation. The segmentation workflow in ITK-SNAP is divided into three logical stages. We notice that the manual pre-segmentation in the first stage, which is performed by applying a smooth threshold, has a significant impact on the final segmentation result. In the following tests, in order to compare the performances of the algorithms, we used the default smooth thresholds for the 1–3 images and manual tuned thresholds for the 4–5 images, which can not generate reasonable results by applying default thresholds. In particular, we set the two-sided thresholds as (418, 703) and (400, 660) for the forth and fifth images, respectively. We also tweaked other major parameters in ITK-SNAP and Seg3D, respectively, for the best segmentation results for these five images. All the three segmentation methods used the same initial labels for each image, as shown in the first column of Fig. 8.

Figure 8 shows the performances of ITK-SNAP, Seg3D and the proposed method on five real brain volumetric MR images with complex image conditions, such as low contrast, intensity inhomogeneity, different level of noise, and weak object boundary. The original low- and high-grade glioma MR images from BRATS12 data sets [86] and the initial labels are shown in the first column. The corresponding ground truths posted with these images are shown in the second column. The segmentation results obtained by ITK-SNAP, Seg3D and the proposed method are plotted on the images in the third, forth and fifth columns, respectively. It can be observed that the segmentation results of the three methods for the first image look similar to the ground truth by visual comparison, showing the capability of these methods in handling intensity inhomogeneity. However, for the second and third images in which the overlaps of the tissue intensity distributions are too large even for human observers, the errors of ITK-SNAP and Seg3D are obvious. By contrast, the proposed method generates visually reasonable segmentation results. For the 4–5 images with weak object boundaries, the leakage issue of ITK-SNAP is serious, while Seg3D and our method generate visually comparative results. The following experiment can demonstrate the significant advantage of our method by quantitative evaluating the segmentation results.

**Fig. 9:**
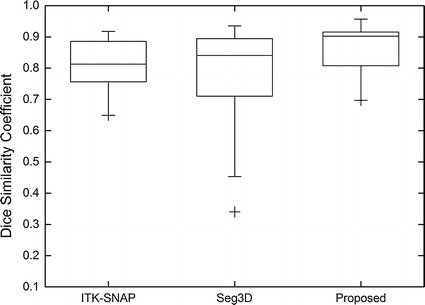
The DSC values of three different segmentation methods on 80 images from BRATS12 data sets. The proposed method achieved more accurate and consistent segmentation results compared with ITK-SNAP and Seg3D

We perform more objective and precise comparison of the three methods in terms of segmentation accuracy by using the DSC as the metric. The DSC values of the three methods are computed against the ground truth, and are provided in Table 2 for the five brain tumor images previously used and Fig. 9 for all the 80 cases from BRATS12 training data sets, respectively. As can be seen, the proposed method achieves more accurate and robust segmentation results. Specifically, the DSC values of ITK-SNAP, Seg3D and the proposed method on the 80 cases of BRATS12 training data sets are 0.8102 ± 0.0718, 0.7665 ± 0.1586 and 0.8802 ± 0.0595, respectively.

**Table 2:** The DSC values for the segmentation results of the three different methods on five images in Fig. 8

Approach	Images in Fig. 8 in the same order				
	Img. 1	Img. 2	Img. 3	Img. 4	Img. 5
ITK-SNAP	0.8966	0.9054	0.8242	0.8025	0.8974
Seg3D	0.9024	0.9032	0.8827	0.8744	0.9074
Proposed	0.9543	0.9149	0.9097	0.9108	0.9242

**Fig. 10:**
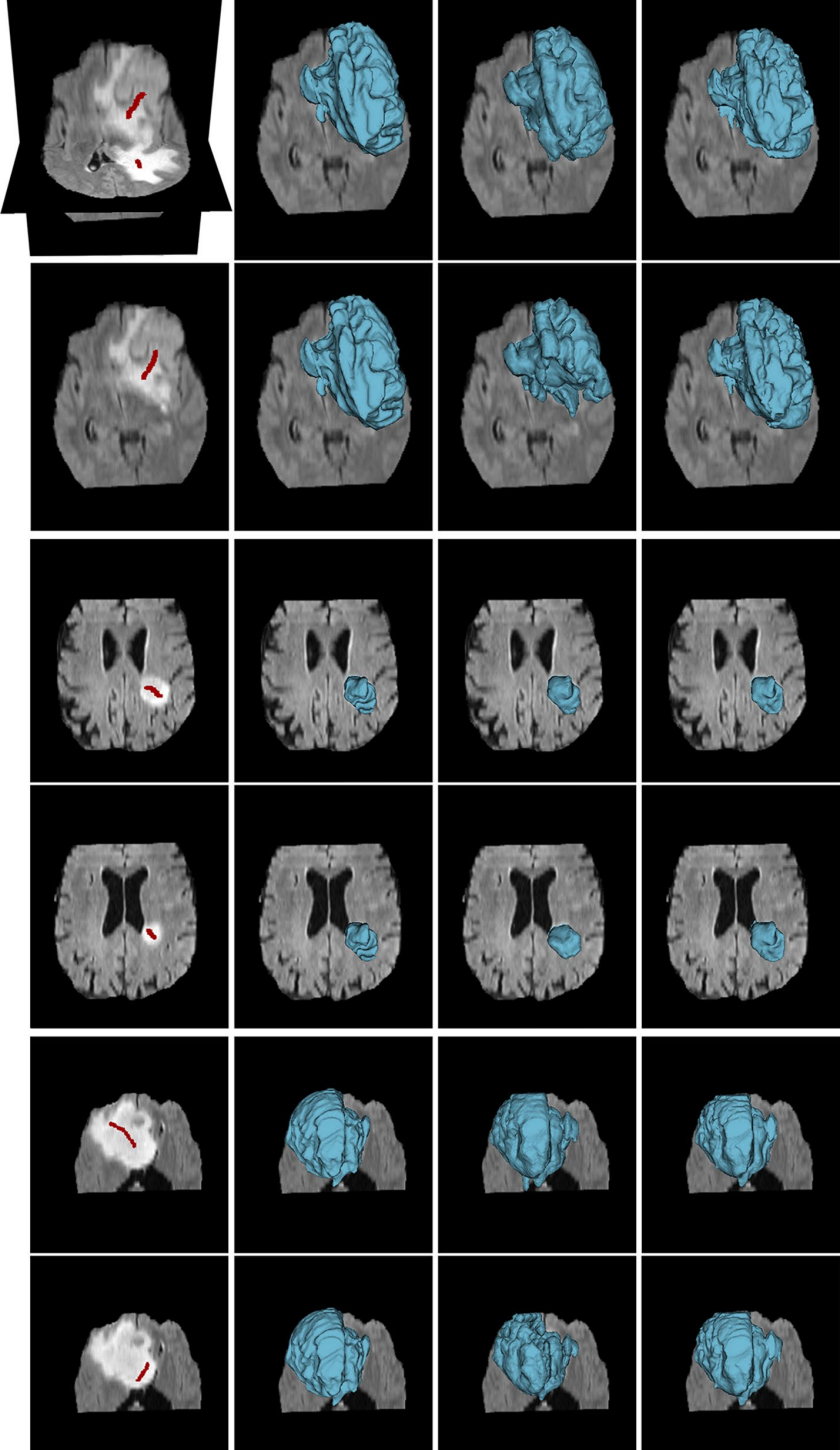
Comparison of our method with the LRS model in 3D Slicer on three groups of brain tumor volumetric MR images. *Column 1* Different initial seeded labels (*red strokes*) are drawn on the original images; *column 2* ground truth; *column 3* the LRS model; *column 4* the proposed method

For comparison with 3D Slicer, we used the “Robust Statistics Segmenter” module embedded in 3D Slicer, which is a fast implementation of the LRS model [70]. Figure 10 shows the results of comparison with the LRS model. We notice that the segmentation result of LRS model is somewhat sensitive to the locations of the initial seeds/strokes and the choice of three major parameters: approximate volume *AV*, intensity homogeneity *IH* and boundary smoothness *BS*. We have carefully initialized the seeds and tweaked these three parameters, which are represented as a triple (*AV*, IH, BS) = (400, 0.6, 0.07), (200, 0.1, 0.2) and (900, 0.05, 0.02) in the order from left to right, for the best segmentation results for these three images [86] in rows 1, 3 and 5 in Fig. 10. Both the images and the parameters in rows 2, 4 and 6 are the same as that in rows 1, 3 and 5 in the same order. In order to compare the robustness of the two methods to seed initialization, we roughly placed the seed labels in the images in rows 2, 4 and 6. Our model and the LRS model use the same initial seeds in each row.

By visual inspection, the proposed model and the LRS model achieve comparable results with sophisticated seed placement (see rows 1, 3 and 5 in Fig. 10), while the proposed model exhibits higher segmentation accuracy and robustness than the LRS model dose with rough initial seeds (see rows 2, 4 and 6 in Fig. 10). To qualitatively compare the results of different models, the DSC values of these two models are computed against the ground truth, and are provided in Table 3 for the images in Fig. 10, which clearly demonstrates the advantage of our model over the LRS model.

**Table 3:** The DSC values for the segmentation results of the two different methods on images in Fig. 10

Approach	Three images with different seed placement in Fig. 10 in the same order					
	Img. 1		Img. 2		Img. 3	
	Sophisticated initial seeds	Rough initial seeds	Sophisticated initial seeds	Rough initial seeds	Sophisticated initial seeds	Rough initial seeds
The LRS model	0.8890	0.7165	0.8636	0.6735	0.9263	0.8095
Proposed	0.9051	0.9042	0.8534	0.8396	0.9286	0.9296

The proposed model is also superior in terms of user interaction. Both the proposed model and the LRS model are semi-automatic methods. Given a small number of user-specified seed labels, the rest of the image can be segmented automatically. The main portion of user interaction in these two models is selecting the initial seeds. Due to the significant advantage of the proposed method in terms of robustness to contour initialization, which has been demonstrated by the experimental results shown in Fig. 10 and Table 3, the user initialization scheme of the proposed method can be easier to implement than that of the LRS model.

In is necessary to note that although a large variety of imaging modalities with different types of biological information can be used for improving the accuracy of tumor delineations, a combination of imaging modalities is beyond the scope of this paper. Therefore, we do not claim to have the best multimodal segmentation, but instead present a promising single modal image segmentation approach which gives some methodological improvements of the field.

## Discussion

### Impact of the parameters

There are two scale parameters *σ* and *η* in the proposed model which control the scales of volumes in calculating the robust statistics features and the fitting energies, respectively. Although we set fixed values for both the two scale parameters in this work, different scale parameters can be incorporated into the proposed model to further improve the performance. In order to examine the influence of these two scales parameters on the performance of the proposed model, we segment the severe contaminated volumetric MR image used in the last row in Fig. 3 with our method using three different values for the two scale parameters. Table 4 shows the HD values of segmenting the WM and GM in the last row in Fig. 3, under various scale parameters *σ* and *η*, with respect to the ground truth.

**Table 4:** The HD values for different combinations of the scale parameters

	WM			GM		
	*η* = 3.0 (mm)	*η* = 6.0 (mm)	*η* = 10.0 (mm)	*η* = 3.0 (mm)	*η* = 6.0 (mm)	*η* = 10.0 (mm)
*σ* = 0.5	10.06	12.19	17.54	10.12	12.41	18.2
*σ* = 1.0	8.65	10.7	16.93	8.34	11.62	18.04
*σ* = 3.0	11.24	15.32	24.16	11.17	17.25	26.93

Intuitively, a smaller scale parameter *η* for the fitting energy can make the algorithm produce more accurate segmentation results in presence of intensity inhomogeneity, while a larger *σ* for the robust statistics feature increases the robustness of the proposed method in terms of noise. For various types of data, we examined and found that the two scale parameters *η* and *σ* need to be adjusted in the range from 3.0 to 30.0 according to the degree of intensity inhomogeneity, and from 0.5 to 5.0 according to the level of noise, respectively.

The parameter *ω* is a constant, which controls the influence of the user specified regions and the local volumes around the center voxels on the two sides of the evolving contours. With a larger parameter *ω*, the evolution of the contours in our method would be dominated by the native characters of the image like in the RSF model. In fact, the RSF model can be considered as an extreme case of the proposed model for *ω* → 1. This can be seen from the limit of the fitting function *P*_*i*_ in Eq. 9 as *ω* → 1. It can be shown that

22 $$\mathop {\lim }\limits_{\omega \to 1} P_{i} (x) = \int_{{\varOmega_{i} }} {K_{\eta } (x - y)p\left( {\mu_{i} (x) - f(y)} \right)dy} ,\begin{array}{*{20}c} {} & {i = 1,2} \\ \end{array} .$$

The right-hand side in Eq. 22 is the robust statistics in the local volumes, which is in the same form of region-scalable fitting energy in the RSF model, while difference in the usage of local information.

### Computational complexity

The main computational cost in the proposed method is for computing *P*_*i*_ in Eq. 18, *u*_*i*_ in Eq. 19 and $\lambda_{ 1} e_{ 1} - \lambda_{ 2} e_{ 2}$ in Eq. 20. The integrals in the numerators and denominators in these equations are computed for each voxel in the target volumetric image. By factorizing these integrals and merging similar terms, there are totally five integrals left to be computed at each iteration, resulting in segmentation running times of approximately 10 s per iteration on a 256 × 256 × 186 pixel image.

Although the proposed method consists of a few computationally expensive steps, the computational efficiency can be significantly improved by using a narrow band scheme described in [21]. For example, the typical algorithm running time of the narrow band implementation of the proposed method for each case in the BRATS12 training data sets can be within 3 min, which were recorded from our experiments with c++ code run on a Lenovo Thinkpad T520i PC, with Intel Core i3 Processor, 2.30 GHz, 4 GB RAM, with Visual studio 2005 on Windows 7. Indeed, although we have schemes to accelerate the algorithm, their efficiency is never perfect. Sometimes, if the running speed is not fast enough, the parameters of the proposed algorithm may not be tuned with ease via trial-and-error. Therefore, in order to guarantee the scalability of the proposed method to large and growing databases, the computational efficiency need to be improved in the near future.

### Some extensions

Currently we only use certain local robust statistics for image features extraction. However, the proposed volume-scalable method provides a basic scheme into which more sophisticated image features can be incorporated, such as Fourier and wavelet features. Some priori information, such as the shape priors, can also be incorporated into the proposed scheme. Combined with the fitting energy functional, this is expected to further improve the accuracy and robustness of the proposed method. Moreover, although the proposed method has been accelerated by a narrow band scheme, the computational efficiency can be further improved by using GPUs or by sparse field level set method. Due to the space limit, details of the above extensions are not included in this paper.

## Conclusion

In contrast to traditional methods, in this paper we presented a semi-automated volume-scalable 3D ACM for segmenting volumetric medical images with complex image conditions. The robust statistics was employed at a controllable scale to extract image information in local volumes. In order to characterize the voxels in the desired objects, a hybrid PDF was proposed according to the local volumes around the intermediate contour and user specified initial seeds. We defined the final energy functional in a volume-scalable manner.

As demonstrated in our experiments, the proposed method can handle intensity inhomogeneity as well as weak object boundary with high level noise in volumetric medical images. The proposed method achieved a high accuracy of 0.9246 ± 0.0068 for WM, 0.9043 ± 0.0131 for liver tumors, 0.8802 ± 0.0595 for brain tumors, etc., measured by DSC value for the overlap between the algorithm one and the ground truth. With the simplified initialization of the active contour, the proposed model held high robustness to initialization. The average DSC value for the ten segmentation results with different initializations is 0.9216 ± 0.0037. Comparative experiments shown desirable performance of the proposed method over several well-known segmentation methods. All of these proven that the application of our proposed volumetric medical image segmentation method can clearly benefit the development of image-based diagnosis/surgery systems.

## Abbreviations

MR: magnetic resonance
CT: computed tomography
WM: white matter
EM: expectation–maximization
RF: random forest
VSMEAN: volume-scalable intensity mean
VSIQR: volume-scalable inter-quartile range
WIV: weighted intensity variance
HD: Hausdorff distance
DSC: Dice similarity coefficient
ACM: active contour model
ANN: artificial neural network
PDF: probability distribution function
RSF: region-scalable fitting
LRS: local robust statistics
VSRF: volume-scalable robust statistics fitting
INU: intensity non-uniformity
STD: standard deviation
SBGFRLS: selective binary and gaussian filtering regularized level set
GM: gray matter
